# Dengue fever as a reemerging disease in upper Egypt: Diagnosis, vector surveillance and genetic diversity using RT-LAMP assay

**DOI:** 10.1371/journal.pone.0265760

**Published:** 2022-05-02

**Authors:** Mona Gaber, Alzahraa Abdelraouf Ahmad, Asmaa M. El-Kady, Mohammed Tolba, Yutaka Suzuki, Shereen M. Mohammed, Nahed Ahmed Elossily

**Affiliations:** 1 Department of Parasitology, Faculty of Medicine, Assiut University, Assiut, Egypt; 2 Department of Parasitology, Faculty of Medicine, South Valley University, Qena, Egypt; 3 Department of Computational Biology and Medical Sciences, University of Tokyo, Kashiwanoha, Kashiwa, Chiba, Japan; 4 Department of Microbiology and Immunology, Faculty of Medicine, Assiut University, Assiut, Egypt; Faculty of Science, Ain Shams University (ASU), EGYPT

## Abstract

**Background:**

The recent increase in dengue virus (DENV) outbreaks and the absence of an effective vaccine have highlighted the importance of developing rapid and effective diagnostic surveillance tests and mosquito-based screening programs. To establish effective control measures for preventing future DENV transmission, the present study was established to identify the main mosquito vector involved in the dengue fever (DF) outbreak in Upper Egypt in 2016 and detect the diversity of dengue virus serotypes circulating in both humans and vectors.

**Methods:**

We investigated the prevalence of DENV infection and circulating serotypes in the sera of 51 humans clinically suspected of DF and 1800 field-collected *Aedes aegypti* adult female mosquitoes grouped into 36 pooled samples. Both DENV non-structural protein (NS1) immunochromatographic strip assay and loop-mediated isothermal amplification (LAMP) were used for screening.

**Results:**

Overall, the rate of DENV infection in both human sera and pooled mosquito homogenate was 33.3%, as revealed by rapid dipstick immunochromatographic analysis. However, higher detection rates were observed with RT-LAMP assay of 60.8% and 44.4% for humans and vector mosquitoes, respectively. DENV-1 was the most prevalent serotype in both populations. A combination of two, three, or even four circulating serotypes was found in 87.5% of total positive pooled mosquito samples and 83.87% of DENV-positive human sera.

**Conclusion:**

The study reinforces the evidence of the reemergence of *Aedes aegypti* in Upper Egypt, inducing an outbreak of DENV. Mosquito-based surveillance of DENV infection is important to elucidate the viral activity rate and define serotype diversity to understand the virus dynamics in the reinfested area. Up to our knowledge, this is the first report of serotyping of DENV infection in an outbreak in Egypt using RT-LAMP assay.

## Introduction

Dengue virus (DENV) is one of the most prevalent mosquito-transmitted arboviruses, which is widely distributed in many tropical and subtropical countries in Southeast Asia, the Pacific, the Americas, and rural areas in Africa [1]. It belongs to the family Flaviviridae and causes human infections via bites of infected female mosquitoes of *Aedes aegypti* or *Aedes albopictus* [2, 3]. A recent estimate indicated that 2.5 billion people were at risk of infection with overall 390 million infections per year, approximately 500,000 hospitalizations, and 25,000 deaths every year [4]. However, the emergence of new epidemics has represented a significant global health problem since the early 20th century [5].

Symptomatic DENV infection was categorized by the WHO as undifferentiated fever, dengue fever (DF), dengue hemorrhagic fever (DHF), or dengue shock syndrome (DSS) [6]. Infection could be due to one of four serologically related but antigenically distinct DENV serotypes (DENV-1 to DENV-4). Based on phylogenetic analysis of the DENV envelope glycoprotein (E), these serotypes could be further subdivided into distinct genotypes [7, 8]. Human infection with a single serotype induces lifelong protective immunity against homologous challenge, but reinfection with heterologous serotypes could produce partial or transient protection [9].

More than 3500 mosquito species have been identified all over the world [10]. *Aedes aegypti* mosquito is identified as the main vector of many serious and medically important arbovirus-caused diseases, such as yellow fever, dengue, Zika, and Chikungunya [11].

Recently, the distribution of DENV infection has expanded markedly, particularly in the Americas, South Asia, and the Western Pacific [6]. However, in recent years, DENV outbreaks were also recorded in the Eastern Mediterranean, Saudi Arabia, and Yemen [12–14] In Egypt, reported cases of dengue declined after 1940, which is attributed to effective vector control measures including the extensive use of dichlorodiphenyltrichloroethane (DDT), leading to the complete eradication of *Aedes aegypti* mosquitoes [12]. Only sporadic studies have reported the reemergence of *Ae*. *aegypti* in Egypt in some southern governorates [15–17]. Studies previously conducted in Assiut Governorate revealed the presence of other *Aedes* species such as *Ae*. *detritus* and *Ae*. *caspius* [18].

A recent DF outbreak was documented by the WHO in Dayrout District of Assiut Governorate, Upper Egypt. Cases were admitted to Dayrout Fever Hospital with acute febrile illness, but there were no serious complications or fatalities. The diagnosis was confirmed by ELISA and PCR, revealing DENV type I. In addition, field-based vector surveillance revealed the presence of *Ae*. *aegypti* larvae and adult mosquitoes at the patients’ dwellings [19–21].

Generally, rapid and efficient diagnosis of infection is critical for proper management and appropriate control of the disease. DF surveillance in humans in most resource-limited countries mainly depends on serological detection of DENV-specific antibodies. Virus isolation, which is the gold standard test for diagnosis of DENV infection, and the RT-polymerase chain reaction (RT-PCR) are highly sensitive and definitive for diagnosing dengue fever, but they are time-consuming, require trained staff, and sophisticated equipment [22]. So, they still have limited applicability in the surveillance field and are not appropriate to use in resource-limited settings besides they cannot differentiate between virus serotypes [23, 24].

Recently, reverse-transcription loop-mediated isothermal amplification (RT-LAMP) was introduced to overcome the limitations of PCR. LAMP is a single-step test used for the amplification of ribonucleic acid (RNA). It includes a combination of the two enzymes, Bst (*Bacillus stearothermophilus*) DNA polymerase and reverse transcriptase, and specific primers designed to recognize six distinct regions of a target sequence. The LAMP technique is sensitive, specific, cost-effective, less time-consuming, and easily interpreted [25, 26].

For the successful surveillance of dengue disease, there is a need for complementary epidemiological studies on virus-transmitting mosquitoes including; viral sequences, mosquito abundance, viral load, and the prevalence of different viral serotypes. In addition, field-collected mosquito surveillance is valuable for tracking virus activity and endorsing control measures [27, 28]. Therefore, there is a need to develop sensitive, specific, cheap, and effective vector surveillance techniques for initial diagnosis and early disease control.

The current study was performed to investigate the reintroduction of *Aedes aegypti* mosquitoes producing a dengue outbreak in Upper Egypt governorates, diagnose DENV infection, and determine the diversity of circulating serotypes. We further highlight the importance of field-based studies for identifying and characterizing DENV serotypes in naturally infected mosquitoes concurrent with human dengue infection for inferring local viral activity in a certain period and locality using RT-LAMP assay.

## Material and methods

### Study area

This study was conducted to investigate the DENV outbreak that occurred during the period from June to December 2016 in three Egyptian governorates: Assiut Governorate (Dayrout and El Kosia Districts), Qena Governorate (Qena District), and Red Sea Governorate (Safaga City).

### Ethical consideration

The study protocol was reviewed and approved by the Ethical Committee of the Faculty of Medicine, Assiut University. Informed consent was obtained from all participants (IRB code:1630083).

### Recruitment and sample collection

#### 1. Human sample collection

A total of 51 serum samples were collected from patients admitted to fever hospitals at the three Egyptian governorates mentioned above and suspected of being infected with DENV. The inclusion criteria of the patients recruited in the study were as follows: fever of unknown origin from 2 to 7 days before hospital admission, headache, joint pain, back pain, retroorbital pain, and skin rash in accordance with the WHO guidelines for suspected Dengue cases [6].

#### 2. Mosquito collection

Approximately 3800 adult mosquitoes were collected from 104 households and 27 outdoor localities from the same residences of the included patients. Resting mosquitoes were captured using a Prokopack aspirator [29]. The collected mosquitoes were stored on dry ice and transferred to the laboratory of the Parasitology Department, Faculty of Medicine, Assiut University, for examination.

Mosquitoes were identified according to morphological identification keys [30]. Approximately 1000 adult mosquitoes were identified as *Culex pipiens* and *Culex perexiguus*. A total of 2800 mosquitoes that were phenotypically identified as *Ae*. *aegypti* in the patient’s settings were sorted according to sex and counted. There were 1800 female *Ae*. *aegypti* mosquitoes (64.3%), the average mosquito density was 14.1 *A*. *aegypti* females per aspiration site. They were divided into 36 pooled samples each containing about 50 adult females. *Culex* spp. and *Ae*. *aegypti* male mosquitoes were not processed further in the study [31]. Wings and legs of the female mosquitoes were removed. In each pool, heads, thoraxes, and abdomens of the mosquitoes were collected and macerated in 300 ml of PBS, followed by thorough homogenization using a Retsch mixer mill MM 200 (Germany). Samples were stored at −80°C until use.

The details of the collected human sera and mosquito samples in relation to the collection sites were illustrated in Table 1.

**Table 1:** Descriptive data on the number and sites of the recruited samples in the study.

	Human samples		Mosquitoes samples					
Governorate	Hospitals	No. of patients	Collection site		Mosquitoes collected			No. of pools
			Households	Outdoor	*C*. *Pipiens* and *C*. *Perexiguus*	*Ae*. *Aegypti* Male	*Ae*. *Aegypti Female*	
**Assiut**	Dayrout fever hospital	5	36	9	500	300	100	2
	El Kosia fever hospital	3						
**Qena**	Qena fever hospital	16	34	9	200	100	500	10
**Red Sea**	Red Sea fever hospital	27	34	9	300	600	1200	24
**Total**		51	104	27	1000	1000	1800	36

### Detection of dengue NS1 Ag in human samples and *Aedes aegypti* pools by immunochromatographic strip test

The dengue NS1 Ag rapid test is an immunochromatographic strip test used for the early diagnosis of dengue infection, even before the presence of IgM or IgG antibodies [32]. It is based on the qualitative detection of non-structural protein 1 (NS1) of DENV. Dengue NS1 Ag strip kit (product code: 70700, Bio-Rad Laboratories, Marnes-la Coquette, France) was used following the manufacturer’s protocol. Briefly, the test was performed for human serum samples and mosquito homogenate. The tested sample (50 μl) was added to the test tube with a single drop of migration buffer, provided with the kit. The test strip was placed in the tube and left for 15 min. Results were obtained after 15 min and could be prolonged for another 15 min for doubtful results. Interpretation of results depended on the appearance of blue color at the Test Line (T) and the Control Line (C) indicating a positive result. While the appearance of (C) line only indicated a negative result. The absence of any bands indicated invalid test

### Detection and serotyping of DENV in both human serum samples and mosquito pools using RT-LAMP

To quantify the viral load, RNA was extracted from *Aedes aegypti* homogenate pools and patient sera using the QIAamp viral RNA mini kit (QIAGEN, Hilden, Germany), in accordance with the manufacturer’s instructions. The extracted RNA was eluted in 100 μl of elution buffer and stored at −70°C until analysis.

#### 1. Design of dengue virus serotype-specific RT-LAMP assay primers

Four DENV serotype-specific RT-LAMP primer sets were designed using the LAMP primer design support software program (Net Laboratory, Japan; http://venus.netlaboratory.com), in accordance with a previous study [23]. Six different primers for each serotype were designed from the 3′ noncoding region (NCR) of the DENV. In each primer set, there are two outer primers (F3, forward outer primer; and B3, backward outer primer), two inner primers (FIP, forward inner primer; and BIP, backward inner primer), and two loop primers (FLP, forward loop primer; and BLP, backward loop primer), which are used to accelerate the amplification reaction. These primers were designed to recognize eight distinct regions on the target DENV sequence. The primers were selected based on criteria described previously [33]. Details of the primer sequences used in the study are described in Table 2.

**Table 2:** Primer sequences used for RT-LAMP targeting DENV serotypes.

Virus serotype	Primer	Sequences (5ˋ ˗ 3ˋ)	bp
**DEN-1**	**F3**	GAGGCTGCAAACCATGGAA	**199 bp**
	**B3**	CAGCAGGATCTCTGGTCTCT	
	**FIP**	GCTGCGTTGTGTCTTGGGAGGTTTTCTGTACGCATGGGGTAGC	
	**BIP**	CCCAACACCAGGGGAAGCTGTTTTTTTGTTGTTGTGCGGGGG	
	**FLP**	CTCCTCTAACCACTAGTC	
	**BLP**	GGTGGTAAGGACTAGAGG	
**DEN-2**	**F3**	TGGAAGCTGTACGCATGG	**211 bp**
	**B3**	GTGCCTGGAATGATGCTG	
	**FIP**	TTGGGCCCCCATTGTTGCTGTTTTAGTGGACTAGCGGTTAGA GG	
	**BIP**	GGTTAGAGGAGACCCCCCCAATTTTGGAGACAGCAGGATCTCTG	
	**FLP**	G GATCTGTAAGGGAGGGG	
	**BLP**	GCATATTGACGCTGGGA	
**DEN-3**	**F3**	GCCACCTTAAGCCACAGTA	**218 bp**
	**B3**	GTTGTGTCATGGGAGGG	
	**FIP**	TGGCTTTTGGGCCTGACTTCTTTTTTGAAGAAGCTGTGCAGCCTG	
	**BIP**	CTGTAGCTCCGTCGTGGGGATTTTCTAGTCTGCTACACCGTGC	
	**FLP**	CCTTGGACGGGGCT	
	**BLP**	GGAGGCTGCAAACCGTG	
**DEN-4**	**F3**	CTATTGAAGTCAGGCCAC	**229 bp**
	**B3**	ACCTCTAGTCCTTCCACC	
	**FIP**	TGGGAATTATAACGCCTCCCGTTTTTTCCACGGCTTGAGCAA ACC	
	**BIP**	GGTTAGAGGAGACCCCTCCCTTTTAGCTTCCTCCTGGCTTCG	
	**FLP**	GGCGGAGCTACAGGCAG	
	**BLP**	TCACCAACAAAACGCAG	

#### 2. Amplification of DENV serotypes by RT-LAMP assay

The RT-LAMP assay was carried out in a total reaction volume of 25 μl using a Loopamp RNA amplification kit (RT-LAMP) (Eiken Chemical Co. Ltd., Japan). The reaction mixture included 6 μl of the primer mix (5 pmol each of F3 and B3 outer primers, 40 pmol each of FIP and BIP inner primers, 20 pmol each of loop primers F and B), 18 μl of LAMP buffer mix (0.1% TritonX, 25× LAMP buffer, 100 mM MgSO_4_, 25 mM dNTPs, 3 mM hydroxynaphtolblue, 0.35% v/v GelGreen, 0.1 μl of RNase inhibitor, 1 μl of Bst DNA polymerase (New England Biolabs), 0.03 μl of AMV reverse transcriptase enzyme (Nippongene), 2 μl of 2 M trehalose, and 1 μl of template RNA of the samples. Each sample was applied to four tubes, each of which contained the primer mixture for a specific serotype. The reaction mixtures were incubated at 63°C for 60 min in a Loopamp real-time turbidimeter (Teramecs, Japan). Positive and negative controls were included in each run. Precautions to prevent cross-contamination were carried out.

#### 3. Analysis of the RT-LAMP assay

- **Naked eye visualization**: After incubation of the samples, the precipitate was deposited in the bottom of the tubes by a pulse spin, followed by inspection with the naked eye for white turbidity. The precipitate was checked under natural daylight as well as under a UV transilluminator (302 nm).
- **Agarose gel electrophoresis**: For agarose gel electrophoresis, 10 μl aliquots of the RT-LAMP products were electrophoresed in 2% agarose gel in 0.5% Tris-borate buffer (TBE), followed by staining with ethidium bromide, running at 90 V for 90 min, and visualization with a UV transilluminator system at 302 nm.

## Results

### Detection and serotyping of DENV in human samples

Concerning the detection of DENV infection in human samples, 17 out of 51 tested human samples were positive for DENV by rapid dipstick test, representing one-third of the tested sera. Meanwhile, RT-LAMP analysis revealed 31 positive cases (60.8%) (S1 Fig in S1 Raw images), with either a single or combined DENV serotypes. The DENV-1 serotype was dominant (93.5%, 29/31). Mixed infection with more than one serotype was observed in most samples (83.87%, 26/31), while mono-infection with one serotype was observed in 5/31 samples (16.13%). The four DENV serotypes were found together in 13/31 positive samples (41.9%). Moreover, human infection with a single DENV serotype was observed in four samples for the DENV-1 serotype and only one sample for the DENV-2 serotype (Table 3).

**Table 3:** Dengue virus infection and serotype distribution in human samples and mosquito pools per collection site.

Sample	Collection site	No. of samples	DENV positive by Dipstick	DENV positive by LAMP PCR	DENV-1	DENV-2	DENV-3	DENV-4
**Human blood**	**Assiut**	8	2	5	5	3	3	2
	**Qena**	16	6	10	9	7	9	7
	**Red Sea**	27	9	15	15	9	12	11
**Total**		**51**	**17 (33.33%)**	**31 (60.8%)**	**29 (93.55%)**	**19 (61.29%)**	**24 (77.41%)**	**20 (64.52%)**
**Mosquito pools**	**Assiut**	2	0	1	1	1	1	-
	**Qena**	10	4	5	5	2	3	-
	**Red Sea**	24	8	10	9	4	10	3
**Total**		36	12 (33.33%)	16 (44.44%)	15 (93.75%)	7 (43.75%)	14 (87.5%)	3 (18.75%)

Note: The number of DENV serotypes reported in the table represents single infections and mixed infections.

### Detection and serotyping of DENV in mosquito pools

Overall, 12 out of 36 mosquito pools (33.3%) were positive for DENV using rapid dipstick immunochromatographic analysis of NS1 Ag. However, with the RT-LAMP assay, DENV infection was detected in 44.4% (16/36) of pooled mosquito samples (S2 Fig in S1 Raw images). The positive mosquito pools were found to be infected with single or mixed DENV serotypes. DENV-1 was the most common serotype (15 out of 16 positive samples). Mixed infection with two or more serotypes was also detected in 87.5% of the total positive pooled mosquito samples (14/16), including the four DENV serotypes being detected simultaneously in two pools. Serotypes DENV-1 and DENV-3 were identified as a mono-infection in one mosquito pool for each.

A comparison between the two assays used in the study for the detection and serotyping of DENV in consistence with the site of sample recruitment in both human samples and mosquito pools was illustrated in Table 3.

## Discussion

Early studies claimed that *Ae*. *aegypti* had been completely eradicated from Egypt in 1963 [34]. Only a few studies reported the occurrence of this species in different parts of Egypt but did not provide substantial evidence of its reintroduction into the country [19]. The current study provided evidence of the reemergence of *Aedes aegypti* mosquitoes in three Egyptian governorates in Upper Egypt. One of them, Assiut Governorate, was previously documented to have an outbreak of DENV in the year preceding this study, namely, 2015. In this study, 2,800 adult mosquitoes of *Ae*. *aegypti* were captured in fields near the outbreak, providing proof of its reemergence at the studied region in Egypt.

In the last few decades, numerous outbreaks and epidemics of DF or DHF have been reported in many tropical and subtropical countries due to rapid urbanization,increased air travel, suboptimal management of water and solid waste, and gaps in vector control [35–37]. The absence of licensed effective vaccines or specific antiviral treatment restricts proper disease management and increases the urgency of developing and applying effective mosquito control strategies [38].

The surveillance of mosquitoes infected with DENV is an integral component of dengue disease control. It can provide early warning signs for a potential risk of disease transmission and enable monitoring and understanding of virus activity, particularly the predominant circulating serotype [39]. The present study demonstrates the usefulness of field-based screening for DENV for estimating infection rates and identifying circulating DENV serotypes in both field-collected mosquitoes and infected patients.

This study revealed approximately one-third (33.3%) of the recruited dengue-suspected patients and the collected mosquito pools from their residential areas to have a confirmed DENV infection by rapid dipstick immunochromatographic analysis, indicating a high burden of DENV. Our results are consistent with previous studies that reported the high specificity and sensitivity of detection of Dengue NS1 Ag as early as 10 days of infection whether in experimentally infected or naturally infected mosquitoes [40–42]. Also, Mata and colleagues [43, 44] had reported the accuracy and reliability of NS1 antigen rapid immunochromatographic test to confirm DENV infection, especially in the acute phase during DENV-1epidemic, since it was a rapid test, simple to do by minimally trained health personnel that appropriate remote sittings without laboratory infrastructure.

However, the RT-LAMP technique revealed higher detection rates among the human samples and mosquito pools (60.8% and 44.4%, respectively). It is possible that this high infection rate was due to the selection of clinically suspected individuals in an outbreak of DF and the targeted surveillance of mosquito samples which were collected mostly from household settings and around residences of suspected cases of Dengue.

The RT-LAMP assay is a rapid, cost-effective, highly sensitive, and specific technique that has been proven to have greater efficacy in diagnosing DENV than immunochromatographic analysis. This is supported by its ability to detect DENV infection in the early stages of infection when NS1 antigen titers could not be detected and the antibody titers of IgM started to rise [36]. It also has the advantage of being a qualitative and quantitative technique, applicable and convenient for DENV serotyping, especially in low-resource healthcare settings as a valuable test for diagnosing dengue [23].

Remarkably, all DENV serotypes were detected in the study specimens, with DENV-1 being the most common, followed by DENV-3 in both human and mosquito samples. The presence of a mixed infection with two, three, or even four serotypes in both populations was a noteworthy finding that postulates the wide diversity of DENV isolates in the newly emerged locality. Overall, 14 out of 16 DENV-positive mosquito pools and 26 serum samples showed concurrent infection with combined DENV serotypes. Only five human serum samples and two mosquito pools revealed infection with a single serotype, which was predominantly the DENV-1 serotype. The observed genetic diversity of DENV serotypes is most likely due to their high capacity to mutate [8, 45]. These results are consistent with previous reports demonstrating the common occurrence of co-circulating serotypes of the DENV in the wild [46, 47].

The observed variations among DENV populations either in geographically distinct areas or within a particular locality could be attributed to the occurrence of spontaneous viral mutations and the importation of new genotypes from neighboring countries [48]. In addition, several factors may play a role in the wide variation of circulating serotypes in patients and field-caught mosquitoes and the presence of combined infection including multiple feeding behavior of *Ae*. *Aegypti* [49, 50], the high infection rates in humans, especially in naïve population during dengue outbreak, which may induce the emergence of infections with multiple serotypes in both humans and infected mosquitoes [49], the transovarian transmission of DENV in mosquitoes [50], and the presence of asymptomatic undiagnosed cases which may act as a source of infection to the vector [49].

Interestingly, the diversity of DENV serotypes in certain populations was reported in previous studies. Dash et al. (2004) considered DENV-2 and DENV-3 to be the most prevalent genotypes for potential dissemination. Meanwhile, DENV-1 was shown to be the leading serotype in circulation, along with the dominant DENV-2 and DENV-3 serotypes [51]. Consistent with this, Ekwudu et al. (2020) observed higher numbers of vectors bearing DENV-1 and -2 in Colombia. This is reasonable as DENV-1 and -2 serotypes generally replicate at higher RNA levels, showing high infectivity and increased virulence in the *Aedes* mosquito, contributing to their global dissemination and higher disease burden than for other serotypes [38].

Upon mosquito-based screening of DENV in Singapore, the infection rates of *Ae*. *aegypti* and *Ae*. *albopictus* populations were relatively low (1.33% and 2.15%, respectively). However, the dominant dengue serotypes were mainly DENV-1 serotypes, followed by DENV-2 and DENV-3, and then DENV-4 [39], which is in accordance with our results. In addition, in Indian studies in recent decades, the DENV-1 serotype has mostly been isolated [52].

Although the small sample size has limited the scope of this study, up to our knowledge, this is the first report of serotyping of DENV in an outbreak in Egypt. Further studies are needed to confirm the association between the prevailing serotypes in patients and field-collected mosquitoes from the same residence. Correlation of DENV serotype infection with clinical presentations is also recommended. Additionally, more molecular studies on wide geographical regions are crucial to explore virus genotypes and virulence factors in both humans and vectors to provide a better insight into the virus dynamics and distribution.

## Conclusion

The present study documented the occurrence of a second DENV outbreak in three Egyptian governorates in Upper Egypt. Field surveillance near the patients’ residences revealed the reemergence of both larval and adult mosquitoes of *Aedes aegypti*, the main vector for DF transmission, supporting the reestablishment of this mosquito vector species in Egypt. Screening of DENV in humans and mosquito populations revealed high infection rates by RT-LAMP assay, which proved to be more effective than the NS1 immunochromatographic strip assay. Moreover, DENV-1 was the most prevalent dengue virus serotype in both humans and mosquito vectors, with most of the collected samples additionally being infected with other serotypes. The RT-LAMP assay greatly enhances the diagnosis of suspected dengue patients as well as DENV screening of field-collected mosquitoes to evaluate local virus activity at a certain time, understand viral movement, and identify transmission hotspots. Vector control and vaccination programs should be enhanced to prevent future outbreaks.

## Supporting information

S1 Raw images (PDF)

## References

[1] World Health Organisation. Global Strategy for Dengue Prevention and Control 2012–2020. World Heal Organ. 2012; 43.

[2] Guzman MG, Harris E. Dengue. Lancet. 2015;385: 453–465. doi: 10.1016/S0140-6736(14)60572-9 25230594

[3] Barcelos Figueiredo L, Sakamoto T, Leomil Coelho LF, De Oliveira Rocha ES, Gomes Cota MM, Portela Ferreira G, et al. Dengue virus 2 American-Asian genotype identified during the 2006/2007 outbreak in Piauí, Brazil reveals a Caribbean route of introduction and dissemination of dengue virus in Brazil. PLoS One. 2014;9: e104516. doi: 10.1371/journal.pone.0104516 25127366 PMC4134198

[4] Bhatt S, Gething PW, Brady OJ, Messina JP, Farlow AW, Moyes CL, et al. The global distribution and burden of dengue. Nature. 2013;496: 504–507. doi: 10.1038/nature12060 23563266 PMC3651993

[5] Chawla P, Yadav A, Chawla V. Clinical implications and treatment of dengue. Asian Pac J Trop Med. 2014;7: 169–178. doi: 10.1016/S1995-7645(14)60016-X 24507635

[6] World Health Organization. Dengue guidelines for diagnosis, treatment, prevention and control: new edition. World Health Organization; 2009. p. WHO/HTM/NTD/DEN/2009.1. 23762963

[7] Rexliene J, Sridhar J. Genetic diversity and evolutionary dynamics of dengue isolates from India. Virus Dis. 2019;30: 354–359. doi: 10.1007/s13337-019-00538-1 31803801 PMC6864014

[8] Amarilla AA, De Almeida FT, Jorge DM, Alfonso HL, De Castro-Jorge LA, Nogueira NA, et al. Genetic diversity of the e Protein of Dengue Type 3 Virus. Virol J. 2009;6: 1–13. doi: 10.1186/1743-422X-6-1 19627608 PMC2720943

[9] Kurane I, Takasaki T. Dengue fever and dengue haemorrhagic fever: challenges of controlling an enemy still at large. Rev Med Virol. 2001;11: 301–311. doi: 10.1002/rmv.324 11590668

[10] Robert L. Mosquitoes (Culicidae). In: Guide to Entomological Surveillance during Contingency Operations. Walter-Reed Army Inst Res. 2001.

[11] Agramonte NM, Bloomquist JR, Bernier UR. Pyrethroid resistance alters the blood-feeding behavior in Puerto Rican Aedes aegypti mosquitoes exposed to treated fabric. PLoS Negl Trop Dis. 2017;11: e0005954. doi: 10.1371/journal.pntd.0005954 28931018 PMC5624645

[12] Rathor HR. The role of vectors in emerging and re-emerging diseases in the Eastern Mediterranean Region. East Mediterr Heal J. 1996;2: 61–67. doi: 10.26719/1996.2.1.61

[13] Khan NA, Azhar EI, El-Fiky S, Madani HH, Abuljadial MA, Ashshi AM, et al. Clinical profile and outcome of hospitalized patients during first outbreak of dengue in Makkah, Saudi Arabia. Acta Trop. 2008;105: 39–44. doi: 10.1016/j.actatropica.2007.09.005 17983609

[14] Alghazali KA, Teoh B-T, Sam S-S, Abd-Jamil J, Johari J, Atroosh WM, et al. Dengue fever among febrile patients in Taiz City, Yemen during the 2016 war: Clinical manifestations, risk factors, and patients knowledge, attitudes, and practices toward the disease. One Heal. 2020;9: 100119. 10.1016/j.onehlt.2019.100119 PMC7184203 32368608

[15] Heikal OM, El-Bahnasawy MM, Morsy AT, Khalil HH. Aedes aegypti re-emerging in Egypt: a review and what should be done? J Egypt Soc Parasitol. 2011;41: 801–814–801–814. 22435171

[16] Saleh NMK. Aedes mosquito in Aswan Governorate, Egypt. J Egypt Soc Parasitol. 2012;42: 233–238. doi: 10.12816/0006311 22662612

[17] Shoukry NM, Elwan MA, Morsy TA. Aedes aegypti (Linnaeus) re-emerging in southern Egypt. J Egypt Soc Parasitol. 2012;42: 41–50. doi: 10.12816/0006293 22662594

[18] Mostafa AA, Allam KAM, Osman MZ. Mosquito species and their densities in some Egyptian governorates. J Egypt Soc Parasitol. 2002;32: 9–20. 12049273

[19] Abozeid S, Elsayed AK, Schaffner F, Samy AM. Re-emergence of Aedes aegypti in Egypt. The Lancet Infectious Diseases. Elsevier; 2018. pp. 142–143. doi: 10.1016/S1473-3099(18)30018-5 29412959

[20] Ibrahim Hamdi Mohammed.; Khalil Mahmoud; Elsawy Mohamed; Ismail Mohamed Samir.; Alfishawy M. Dengue Fever Outbreak Investigation in Upper Egypt in 2015. Open Forum Infect Dis. 2019;6: S616–S616. doi: 10.1093/ofid/ofz360.1546

[21] Mostafa AA, Rashed ME, Aly NES, Hasan AH, Mikhail MW. Entomological surveillance of Aedes aegypti and arboviruses outbreak of Dengue fever in The Red sea Governorate, Egypt. J Egypt Soc Parasitol. 2019;49: 713–718. doi: 10.21608/jesp.2019.68080

[22] Xi Y, Xu CZ, Xie ZZ, Zhu DL, Dong JM. Rapid and visual detection of dengue virus using recombinase polymerase amplification method combined with lateral flow dipstick. Mol Cell Probes. 2019;46. doi: 10.1016/j.mcp.2019.06.003 31202830

[23] Parida M, Horioke K, Ishida H, Dash PK, Saxena P, Jana AM, et al. Rapid detection and differentiation of dengue virus serotypes by a real-time reverse transcription-loop-mediated isothermal amplification assay. J Clin Microbiol. 2005;43: 2895–2903. doi: 10.1128/JCM.43.6.2895-2903.2005 15956414 PMC1151941

[24] Li S, Fang M, Zhou B, Ni H, Shen Q, Zhang H, et al. Simultaneous detection and differentiation of dengue virus serotypes 1–4, Japanese encephalitis virus, and West Nile virus by a combined reverse-transcription loop-mediated isothermal amplification assay. Virol J. 2011;8: 1–9. doi: 10.1186/1743-422X-8-1 21777455 PMC3149006

[25] Li Y, Fan P, Zhou S, Zhang L. Loop-mediated isothermal amplification (LAMP): A novel rapid detection platform for pathogens. Microb Pathog. 2017;107: 54–61. doi: 10.1016/j.micpath.2017.03.016 28323152

[26] Perera RS, Ding XC, Tully F, Oliver J, Bright N, Bell D, et al. Development and clinical performance of high throughput loop-mediated isothermal amplification for detection of malaria. PLoS One. 2017;12: e0171126–e0171126. doi: 10.1371/journal.pone.0171126 28166235 PMC5293265

[27] Chen CF, Shu PY, Teng HJ, Su CL, Wu JW, Wang JH, et al. Screening of dengue virus in field-caught Aedes aegypti and Aedes albopictus (Diptera: Culicidae) by one-step SYBR green-based reverse transcriptase-polymerase chain reaction assay during 2004–2007 in southern Taiwan. Vector-Borne Zoonotic Dis. 2010;10: 1017–1025. doi: 10.1089/vbz.2008.0069 21128850

[28] Castro MG de, Nogueira RMR, Filippis AMB de, Ferreira AA, Lima M da RQ, Faria NR da C, et al. Dengue virus type 4 in Niterói, Rio de Janeiro: the role of molecular techniques in laboratory diagnosis and entomological surveillance. Mem Inst Oswaldo Cruz. 2012;107: 940–945. doi: 10.1590/s0074-02762012000700017 23147153

[29] Vazquez-Prokopec GM, Galvin WA, Kelly R, Kitron U. A New, Cost-Effective, Battery-Powered Aspirator for Adult Mosquito Collections. J Med Entomol. 2009;46: 1256–1259. doi: 10.1603/033.046.0602 19960668 PMC2800949

[30] Rueda LM. Pictorial keys for the identification of mosquitoes (Diptera: Culicidae) associated with Dengue Virus Transmission. Zootaxa. 2004. doi: 10.11646/zootaxa.589.1.1

[31] Pérez-Castro R, Castellanos JE, Olano VA, Matiz MI, Jaramillo JF, Vargas SL, et al. Detection of all four dengue serotypes in Aedes aegypti female mosquitoes collected in a rural area in Colombia. Mem Inst Oswaldo Cruz. 2016;111: 233–240. doi: 10.1590/0074-02760150363 27074252 PMC4830112

[32] Ramirez A, Moros Z, Comach G, Zambrano J, Bravo L, Pinto B, et al. Evaluation of dengue NS1 antigen detection tests with acute sera from patients infected with dengue virus in Venezuela. Diagn Microbiol Infect Dis. 2009;65: 247–253. doi: 10.1016/j.diagmicrobio.2009.07.022 19733994

[33] Notomi T, Okayama H, Masubuchi H, Yonekawa T, Watanabe K, Amino N, et al. Loop-mediated isothermal amplification of DNA. Nucleic Acids Res. 2000;28: e63–e63. doi: 10.1093/nar/28.12.e63 10871386 PMC102748

[34] Gad AM, Salit A. The Mosquitoes of the Red Sea Area, Egypt. J Med Entomol. 1972;9: 581–582. doi: 10.1093/jmedent/9.6.581 4144019

[35] Guzman MG, Kouri G. Dengue and dengue hemorrhagic fever in the Americas: lessons and challenges. J Clin Virol. 2003;27: 1–13. doi: 10.1016/s1386-6532(03)00010-6 12727523

[36] Sahni AK, Grover N, Sharma A, Khan ID, Kishore J. Reverse transcription loop-mediated isothermal amplification (RT-LAMP) for diagnosis of dengue. Med J Armed Forces India. 2013;69: 246–253. doi: 10.1016/j.mjafi.2012.07.017 24600118 PMC3862550

[37] Gubler DJ. Dengue, Urbanization and globalization: The unholy trinity of the 21 st century. Trop Med Health. 2011;39: 3–11. doi: 10.2149/tmh.2011-S05 22500131 PMC3317603

[38] Ekwudu O, Marquart L, Webb L, Lowry KS, Devine GJ, Hugo LE, et al. pathogens E ff ect of Serotype and Strain Diversity on Dengue Virus Replication in Australian Mosquito Vectors. Pathogens. 2020;9: 1–14. doi: 10.3390/pathogens9080668 32824792 PMC7460537

[39] Kow CY, Koon LL, Yin PF. Detection of dengue viruses in field caught male Aedes aegypti and Aedes albopictus (Diptera: Culicidae) in Singapore by type-specific PCR. J Med Entomol. 2001;38: 475–479. doi: 10.1603/0022-2585-38.4.475 11476326

[40] Tan CH, Wong PSJ, Li MZI, Vythilingam I, Ng LC. Evaluation of the Dengue NS1 Ag strip® for detection of Dengue virus antigen in Aedes aegypti (Diptera: Culicidae). Vector-Borne Zoonotic Dis. 2011;11: 789–792. doi: 10.1089/vbz.2010.0028 21395416 PMC3115461

[41] Muller DA, Frentiu FD, Rojas A, Moreira LA, O’Neill SL, Young PR. A portable approach for the surveillance of dengue virus-infected mosquitoes. J Virol Methods. 2012;183: 90–93. doi: 10.1016/j.jviromet.2012.03.033 22575689

[42] Voge N V., Sánchez-Vargas I, Blair CD, Eisen L, Beaty BJ. Detection of dengue virus NS1 antigen in infected aedes aegypti using a commercially available kit. Am J Trop Med Hyg. 2013;88: 260–266. doi: 10.4269/ajtmh.2012.12-0477 23185074 PMC3583315

[43] Mata VE, Passos SRL, Hökerberg YHM, Berardinelli GM, dos Santos MAB, Fukuoka LVB, et al. Precisão e confiabilidade de um teste imuno-cromatográfico rápido NS1 para diagnóstico DENV-1 no ponto de atendimento e no laboratório. BMC Infect Dis. 2017;17. doi: 10.1186/s12879-017-2679-z 28851293 PMC5576130

[44] Mata VE, De Andrade CAF, Passos SRL, Hökerberg YHM, Fukuoka LVB, Da Silva SA. Rapid immunochromatographic tests for the diagnosis of dengue: A systematic review and meta-analysis. Cadernos de Saude Publica. Fundacao Oswaldo Cruz; 2020. doi: 10.1590/0102-311x00225618 32520127

[45] Angel A, Angel B, Joshi AP, Baharia RK, Rathore S, Joshi V. First study of complete genome of Dengue-3 virus from Rajasthan, India: Genomic characterization, amino acid variations and phylogenetic analysis. Virol Reports. 2016;6: 32–40. doi: 10.1016/j.virep.2016.05.003

[46] Lanciott i R, Lewis J, Gubler D, Trent D. Molecular evolution and epidemiology of dengue-3 viruses. J Gen Virol. 1994;75: 65–75. doi: 10.1099/0022-1317-75-1-65 8113741

[47] Rico-Hesse R, Harrison L, Nisalak A, Vaughn D, Kalayanarooj S, Green S, et al. Molecular evolution of dengue type 2 virus in Thailand. Am J Trop Med Hyg. 1998;58: 96–101. doi: 10.4269/ajtmh.1998.58.96 9452299

[48] Rico-Hesse R. Microevolution and virulence of dengue viruses. Advances in Virus Research 2003 pp. 315–341. doi: 10.1016/s0065-3527(03)59009-1 14696333 PMC3045824

[49] Loroño-Pino MA, Cropp CB, Farfán JA, Vorndam A V., Rodríguez-Angulo EM, Rosado-Paredes EP, et al. Common occurrence of concurrent infections by multiple dengue virus serotypes. Am J Trop Med Hyg. 1999;61: 725–730. doi: 10.4269/ajtmh.1999.61.725 10586902

[50] Thavara U, Siriyasatien P, Tawatsin A, Asavadachanukorn P, Anantapreecha S, Wongwanich R, et al. Double infection of heteroserotypes of dengue viruses in field populations of Aedes aegypti and Aedes albopictus (Diptera: Culicidae) and serological features of dengue viruses found in patients in southern Thailand. Southeast Asian J Trop Med Public Health. 2006;37: 468–476. 17120966

[51] Dash PK, Parida MM, Saxena P, Kumar M, Rai A, Pasha ST, et al. Emergence and continued circulation of dengue-2 (genotype IV) virus strains in Northern India. J Med Virol. 2004;74: 314–322. doi: 10.1002/jmv.20166 15332281

[52] Kukreti H, Chaudhary A, Rautela RS, Anand R, Mittal V, Chhabra M, et al. Emergence of an independent lineage of dengue virus type 1 (DENV-1) and its co-circulation with predominant DENV-3 during the 2006 dengue fever outbreak in Delhi. Int J Infect Dis. 2008;12: 542–549. doi: 10.1016/j.ijid.2008.02.009 18495513

