# Supplementary figures and images for: Dengue fever as a reemerging disease in upper Egypt: Diagnosis, vector surveillance and genetic diversity using RT-LAMP assay
Source: PLoS One. 2022 May 2;17(5):e0265760. doi: 10.1371/journal.pone.0265760 (PMC9060354; doi:10.1371/journal.pone.0265760)

## Supporting information:

### Gel raw images

#### S1 Fig

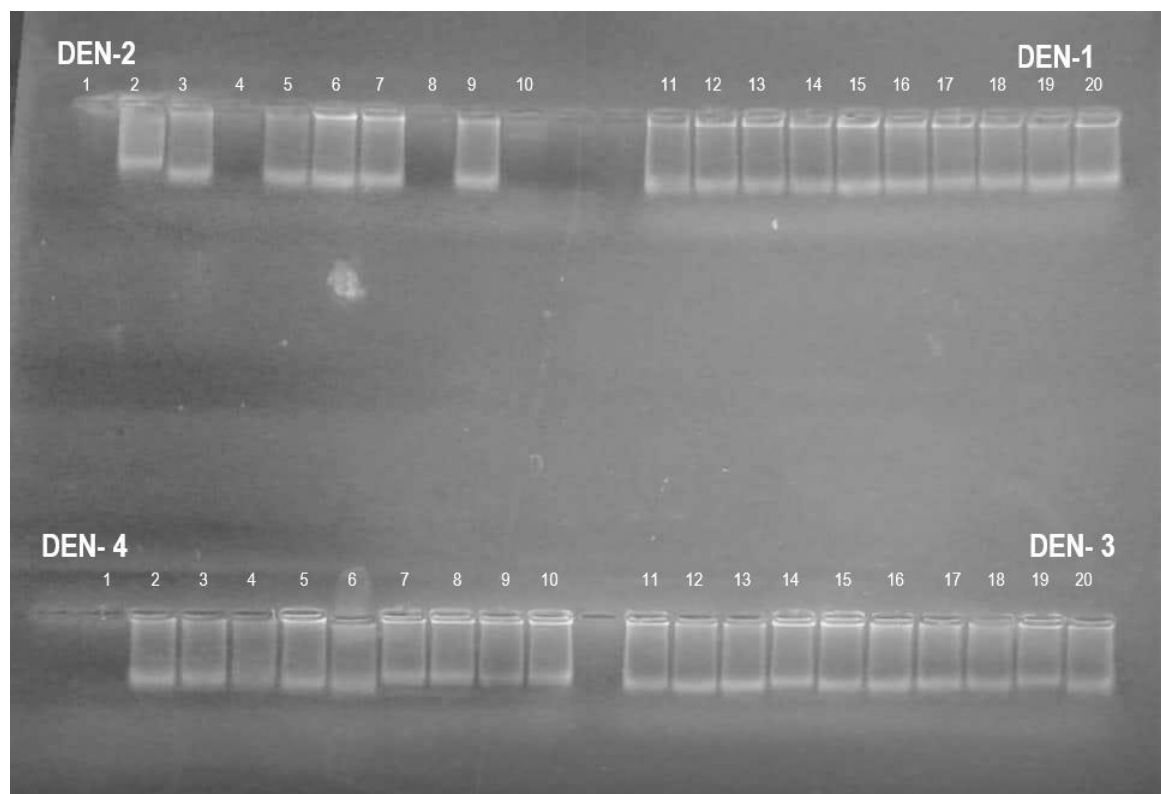

**S2 Fig**

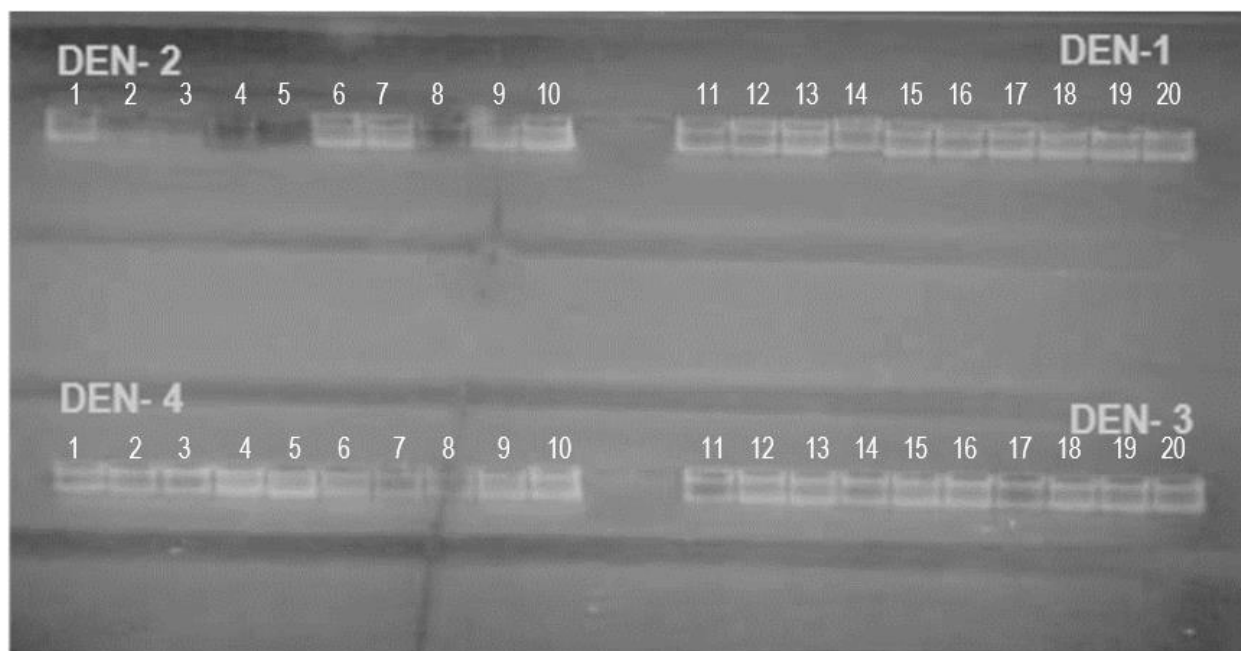

Supplement: S1 Raw images — (PDF) [file pone.0265760.s001.pdf]
